# Causal diagrams in systems epidemiology

**DOI:** 10.1186/1742-7622-9-1

**Published:** 2012-03-19

**Authors:** Michael Joffe, Manoj Gambhir, Marc Chadeau-Hyam, Paolo Vineis

**Affiliations:** 1 Department of Epidemiology and Biostatistics, Imperial College London, London, UK; 2 Department of Infectious Disease Epidemiology, Imperial College London, London, UK

**Keywords:** Epidemiological methodology, Causation, DAGs, Diagrammatic methods, Infectious disease epidemiology models, Web of causation, Instrumental variables, Change models, Feedback

## Abstract

Methods of diagrammatic modelling have been greatly developed in the past two decades. Outside the context of infectious diseases, systematic use of diagrams in epidemiology has been mainly confined to the analysis of a single link: that between a disease outcome and its proximal determinant(s). Transmitted causes ("causes of causes") tend not to be systematically analysed.

The infectious disease epidemiology modelling tradition models the human population in its environment, typically with the exposure-health relationship and the determinants of exposure being considered at individual and group/ecological levels, respectively. Some properties of the resulting systems are quite general, and are seen in unrelated contexts such as biochemical pathways. Confining analysis to a single link misses the opportunity to discover such properties.

The structure of a causal diagram is derived from knowledge about how the world works, as well as from statistical evidence. A single diagram can be used to characterise a whole research area, not just a single analysis - although this depends on the degree of consistency of the causal relationships between different populations - and can therefore be used to integrate multiple datasets.

Additional advantages of system-wide models include: the use of instrumental variables - now emerging as an important technique in epidemiology in the context of mendelian randomisation, but under-used in the exploitation of "natural experiments"; the explicit use of change models, which have advantages with respect to inferring causation; and in the detection and elucidation of feedback.

## Introductory quotes

"Could one of the problems of modern epidemiology ... be that we have drifted back to *a posteriori *methods - fitting black box equations to data, rather than working out predictions from mathematical modeling of underlying processes?" Norman E Breslow, 2003 [1].

"... narrowness of thinking ... pervades much of modern science and leads to inaccurate assessments and prescriptions in many fields. The narrowness itself stems from a perennial challenge with which every scientist must grapple: many phenomena we'd like to understand are highly complex and have multiple, interacting causes." Paul Epstein, 2011 [2].

## The role of causation in epidemiology

Causation is very important in epidemiology. Epidemiologists are traditionally cautious in using causal concepts: the basic method of epidemiology is to observe and quantify associations, whereas causal relationships cannot be directly observed. Causal inference is then a distinct step which is not unproblematic, but which cannot be ignored because the two main purposes of epidemiological evidence are to provide understanding and the basis for intervention, and for both of these it is necessary to know about the causal status of the observed associations.

**Figure 1:**
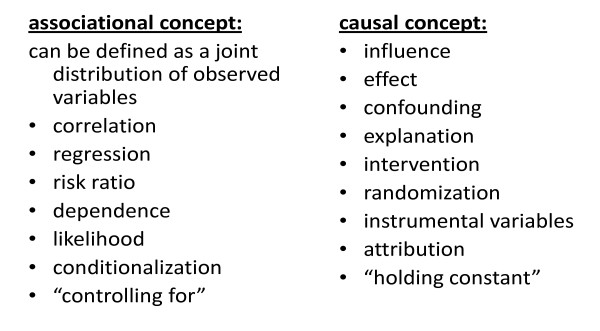
**Pearl: causal & statistical languages**.

Pearl has pointed out that association and causation have entirely separate languages, with terms such as regression, likelihood and "controlling for" belonging to the probabilistic group, as they refer to the observed joint distribution and to ways of manipulating it statistically; whereas terms such as effect, confounding and intervention refer to a causal relationship (Figure 1)[3,4]. In dealing with, for example, confounding, *causal *understanding of the relationship between the variables is indispensable, to avoid adjusting for a covariate that is on the causal pathway. Pearl criticises the typical practice that explicit causal thinking does not occur in the design of the study or the set-up of the analysis, but only afterwards, in interpreting the findings. Thus, assessment of causal inference is left until the Discussion section of a paper, where it is "smuggled in", rather than being part of the Methods section [3,4]. It is more appropriate to develop and use causal language in a rigorous fashion: to be explicit, as well as cautious, in the use of causal concepts.

More abstractly, a causal relationship is one that has a *mechanism *that by its operation *makes a difference *[5,6]. The scientific process of discovery of causal relationships can proceed using either of these features. Epidemiology employs difference-making, i.e. *how much *effect one variable has on another; the other approach, which has a complementary role, is uncovering the mechanism, i.e. explaining *how it exerts that effect *[5,6].

Causal relationships operate over time, so that difference-*making *is distinct from non-causal differences that *exist *between categories of background variables, such as sex differences in disease risk. For example, the higher rate of breast cancer in women than men can be traced to metabolic differences between the two sexes (e.g. high endogenous estrogens in females), which do play a causal role over time. The observed sex difference is due to differences between processes in the two sexes that are themselves causal.

### Causal diagrams

Diagrams consisting of variables connected by arrows or lines are widely used in epidemiology, either formally as in the Directed Acyclic Graph (DAG) literature, or informally as influence diagrams, to depict relationships that are relatively complicated and so are considered to deserve illustrating in this way. In this paper we consider the use of diagrams that denote causation, not merely association: one variable alters the probability, timing, magnitude and/or severity of the next variable; or alternatively they represent the "flow" of, for example, individuals from the status of susceptible to infected and thence to recovered (or dead).

In particular, we review the types of diagram that go beyond the depiction of a single link, e.g. a disease and its proximal causal factor, to focus on a larger *causal system *that is important to health. A "system" in this context is made up of multiple causal relationships, each one of which can be considered as a "link"; and each of the links is considered potentially important, as it could influence how the system as a whole behaves. Because it can be difficult to envisage such multiple links intuitively, and in more complicated cases errors are likely, diagrams are very valuable in showing the inter-relationships. Some of these uses are already well established, especially in infectious disease epidemiology, but we believe that this perspective could be further developed in epidemiology more generally - what could be called "systems epidemiology", by analogy with the recent development of systems biology (see below). Such causal systems could include biochemical pathways, e.g. in relation to biomarkers, or the social/environmental context in which people live that could affect their disease risk.

### Directed Acyclic Graphs (DAGs)

The use of DAGs has gained increasing recognition within epidemiology in recent years, following the work of Pearl, Robins, Greenland and others [3][4][7][8][9][10][11][12][13][14][15][16]. DAGs are simple to use, and in addition it has been shown that if certain simple rules are followed, they provide a rigorous guide to such issues as confounding and selection effects. In general, the procedures associated with DAGs correspond to traditional statistical methods, including informal "rules of thumb" such as not adjusting for a covariate that is on the causal pathway, but they are less error-prone in complicated situations.

**Figure 2:**
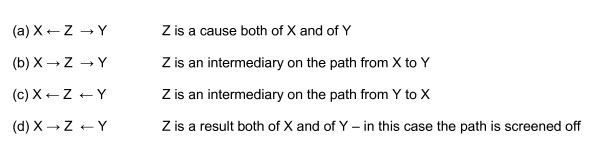
**DAGs representing the relationship of the variables X, Y and Z**.

DAGs are composed of variables connected by arrows (sometimes called directed "edges"), but it is not always clear when these are intended to denote a causal relationship or only a probabilistic one. Figure 2 shows four DAGs that represent ways in which the variables X, Y and Z can be related. In the first three of these, the probabilistic interpretation is identical: all can be described as "X is independent of Y given Z" - but they have totally different causal interpretations, as suggested by the direction of the arrows [17]. Only in the fourth DAG, (d), is the probabilistic interpretation different, because the two arrows pointing at Z indicate that the path is blocked or "screened off" [14]. In addition, some DAG practitioners use inductive procedures involving algorithms to try and derive causal structure directly from the data, rather than empirically testing a hypothesised structure that is constructed a priori [18]; the merits of this approach are controversial [17], a discussion that is beyond the scope of this paper.

Furthermore, the DAG tradition has its limitations: once one goes beyond the technical issues of inferring the causal status of a particular observed association, other considerations come into play. These require the use of other diagram-associated methods, including the modelling of infectious disease outbreaks with differential equations, fitting statistical models to causal networks, and analysing systems characterised by feedback. The wider properties of such systems are scientifically and practically important, yet are insufficiently appreciated in most of epidemiology.

### Systems epidemiology and the use of diagrams

In this paper, we discuss different types of causal system that are relevant to epidemiology: models of infectious disease transmission, in which the human population is located within a broader system with which it interacts; models that integrate the emission and dispersion of pollutants with their impacts on health; and the relationship of social factors to specific risk factors and to selection effects. We describe how diagrams can be employed to improve the analysis of such systems, and in the course of doing so we note that generic properties of the systems can be observed that are independent of the specific content, even though the diagrams themselves have been constructed solely from empirical evidence - no structure has been imposed on them.

We draw on a number of traditions that have analysed systems and/or that have used causal diagrams. The most important of these are:

• path diagram analysis, which was devised by the geneticist Sewall Wright but which has mainly been employed in quantitative social science analysis, and

• the similar but more general method of structural equation modelling, which also systematically analyses measurement error [19] - including the use of latent variables that represent theoretical constructs, estimated from several measured variables;

• econometrics, in which the structure of a system is represented by an equation for each link, albeit without the systematic use of causal diagrams [20];

• diagrams of metabolic pathways in biochemistry;

• the tradition of infectious disease epidemiology modelling [21], which is based on demographic and ecological models involving the relationship between different species;

• a group of traditions in systems modelling, including cybernetics, dynamical systems modelling, and system dynamics [22], as well as open systems theory [23].

## Modelling the larger system

### Models and diagrams in infectious disease epidemiology

In 1897, Ronald Ross established that malaria is spread by the *Anopheles *mosquito, and subsequently received the second Nobel prize for medicine. He then defined a mathematical model describing the time dependent dynamics of infection and recovery in human and mosquito populations. The major terms in the differential equations describing this human-mosquito-parasite ecology were (unless otherwise stated, these terms are numbers per unit time): the number of newly infected humans arising due to bites from infected mosquitoes, the number of new mosquito infections due to biting infected humans, and the rate of recovery of both humans and mosquitoes from infection [24].The explicit expression of these differential equations as an a *priori *model - i.e. a model in which the sole causative agent of disease was assumed from outset to be the protozoan parasite, which was acquired by mosquito biting - led to the startling conclusion that there existed a critical value for the number of mosquitoes per person that needed to be present in order to allow the parasite to persist locally. Ross estimated this critical number of mosquitoes per person to be 40 - implying that *Anopheles *did not need to be eradicated for the disease to die out [1].

Ross reached this conclusion by modelling the whole system: the human population within its environment. It was built on evidence at the individual level, but with some of the (implied) interventions at group or environmental level. His method was not expressed as a diagram, but it represents a sequential causal relationship, the key outcome being whether the number of infected people in one period is higher or lower than that in the previous one. The method was feasible because he focused on the single cause, malaria transmission by mosquito which had already been established, and omitted other relevant factors, e.g. that nutritional status might affect susceptibility.

**Figure 3:**
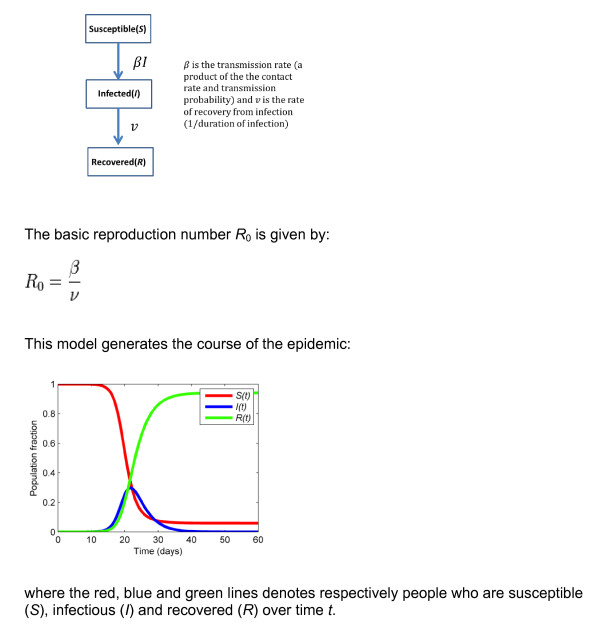
**A flow diagram of the SIR model**.

This pioneering work initiated methodological developments in infectious disease epidemiology, again modelling a system consisting of a human population within its environment [21]. These include compartmental models such as the SIR (Susceptible-Infected-Recovered) model (Figure 3), where the population is sub-divided into states corresponding to observed (or assumed) steps in the disease process. The transitions from one state to the next, represented by differential equations, reflect the causal effects - although causality is not made explicit - with transition probabilities being determined by quantities such as the contact rate, the infection transmission probability and the recovery rate.

Models of this type can be more complex, for example if vector transmission is involved, but the principle remains the same. The equivalent of Ross's critical mosquito density is the basic reproduction number *R*_0_: if is greater than unity, this indicates that the number of new cases in one period is higher than that in the previous one, and therefore that the outbreak can propagate itself; if it is less than unity then the epidemic will fade out. Most such models are deterministic in that they do not consider stochastic causation, but probabilistic elements are increasingly being incorporated [25].

Compartmental models rely on the existence of a single characteristic that can be used to partition the whole population. In the SIR case, the partitioning characteristic is the status of each person with respect to susceptibility and infectiousness. The model is thus mono-causal, neglecting other factors such as nutritional status and the existence of other infections that may influence the recovery rate; models can be modified to take these into account, e.g. stratifying the population into high and low risk groups [26].

### Single-chain models outside infectious disease epidemiology

This approach is no longer used only for modelling infectious diseases. For example, it has been applied to cervical cancer, involving carcinogenic HPV transmission dynamics and the natural history of the disease. It involved comparing scenarios of vaccination against HPV-16, either of 12-year-old girls alone or of both sexes, and of the no-vaccination scenario [27]. Thus, the distinction of infectious and non-infectious disease is somewhat artificial, given that the same modelling methodology can be used in situations where the infectious agent is but one factor contributing to the development of the disease.

More generally, compartmental models can be viewed as a sub-type of diagrammatic models: flow diagrams in which the population is subdivided into ordered states. They are also of interest in chronic disease epidemiology, where they can be used to represent the evolution of health status among known steps of disease progression. These stages can either be observed or hidden (e.g. if the prevalence of the asymptomatic affection cannot be measured) [28,29]. On top of providing a quantification of the impact of risk factors/exposures on the disease risk, these approaches also give an insight into the dynamic of disease progression at the individual level, and at the population level, into the dynamic of the epidemic.

Compartmental models aim at reconstructing the individual or population natural history of the disease progression amongst disease states, based on - potentially longitudinal - exposure or complex mixtures of exposures. Hence, by nature, they incorporate a temporal component in their causal inference, and in accordance with the recently formalised exposome concept [30,31], they allow the disease risk to be driven not only by exposure level itself but also by its evolution in time and by potential temporal patterns in the exposure history.

A similar use of diagrams has long been standard practice in another branch of biology: biochemical pathways. These are flow diagrams in which at each stage, the molecule is modified by an enzyme belonging to that step in the pathway. In practice they are often drawn as cartoons that include also a spatial element, indicating the location of the different chemical processes within the cell.

**Figure 4:**
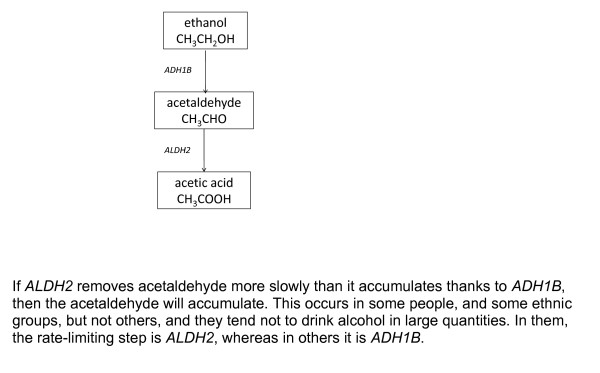
**A flow diagram illustrating a rate-limiting step**.

An example is the metabolism of ethanol (alcohol) via acetaldehyde to acetic acid, which is then metabolised further, yielding carbon dioxide, water and energy (Figure 4). A fundamental concept in biochemical pathways is the rate-limiting step: if conversion of ethanol to acetaldehyde proceeds faster than that of acetaldehyde to acetic acid, but not in the reverse situation, then acetaldehyde accumulates. This depends on the relative speed of the two enzymes, alcohol dehydrogenase IB (class I), beta polypeptide (*ADH1B*) and aldehyde dehydrogenase 2 (*ALDH2*). It so happens that the second of these can be present in different forms, resulting in either faster or slower activity than *ADH1B*, and that this varies with ethnic group. Since acetaldehyde gives rise to unpleasant symptoms (as well as toxicity), this polymorphism explains why some ethnic groups tend to indulge in drinking large quantities of ethanol, whereas others do not.

The situation here is directly analogous to the SIR model, where the tendency of an outbreak to increase or decrease depends on the balance between inflow and outflow. In that situation this balance depends on the force of infection as measured by *R*_0_,: if greater than unity, the outflow is the rate-limiting step and infected individuals will tend to accumulate in the population, like acetaldehyde, and vice versa for values lower than unity. Although both these diagrams have been constructed in radically different contexts, their structure as well as the type of results they provide are comparable, thus highlighting the potential general use of these models. While their formulation is general, the way transitions from one compartment to another are defined is highly specific of the modelled phenomenon. This type of approach relies on the modelling of the whole system rather than focusing on a single link within the system of interest.

**Figure 5:**
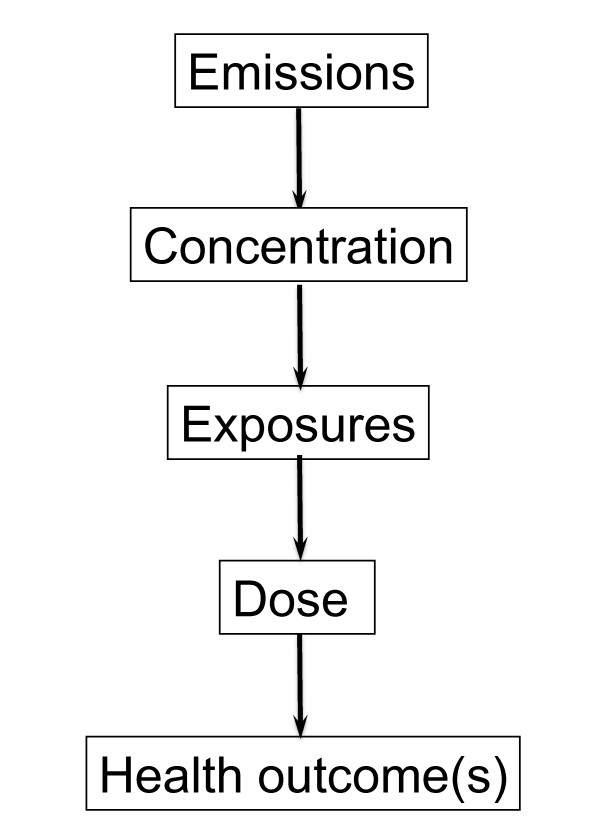
**The full-chain approach in environmental and occupational epidemiology**.

A somewhat similar approach can be used in non-infectious disease epidemiology, for example in environmental and occupational epidemiology, which has increasingly moved towards a study of the whole chain from the existence of a pollutant in the environment, through human exposure, to health outcome (Figure 5)[32]. Here we are concerned with a diagram that is constructed from concepts such as "emissions", "concentration" and "exposures" that correspond to substantive knowledge about how the world works, and which are organised in a form suitable for statistical analysis. Building this type of model requires multidisciplinary collaborative work, e.g. involving hygienists and epidemiologists. Typically the upstream causal processes involve a particular location, so that exposure is ecological, i.e. at group level, whereas for epidemiological analysis the individual level is best, to avoid ecological bias that could result when inference is made from one level to another. This combination of levels is routinely employed in infectious disease epidemiology modelling, and this also integrates disparate types of information, e.g. biological, psychosocial and socioeconomic, as well as medical interventions (e.g. immunisation). More generally, the perspective of modelling the whole system fits with the perception that more attention should be paid to "causes of causes" [33], not only to proximal causes.

### Multiple causation: diagrams with multiple and branching chains

The models considered so far have been concerned with only one causal pathway. However, epidemiology of non-infectious diseases usually deals with a situation of multiple causation, in which all (or most) links are analysed as stochastic - there are no necessary or sufficient causes, and Koch's postulates do not apply. Under such conditions, diagrammatic models are no longer confined to a single chain.

It is simple to draw a diagram that contains branches, but this introduces new issues that go beyond the scope of the present paper. In principle, causal diagrams and DAGs can readily cope with multiple causation, but further methodological work is needed on effect modification [34-36].

In social epidemiology, a classic question is, how much of the observed social gradient is mediated by known risk factors? It is possible to investigate this question on the simple assumption that no effect modification or other complicating factor is present, in which case a diagram is probably not necessary. However, such an assumption may not be justified. For example, an econometric analysis of the Whitehall II Study has shown that if allowance is made for selection effects, the findings change. Whilst childhood socioeconomic circumstances are still found to impact on adult health, it emerges that the association of current civil service grade with health status reflects the tendency for healthier people to be promoted. And employment grade is also predicted by childhood socioeconomic position, which thus influences adult health both directly and via job success - for example, promotion is more likely for taller people, and height is an indicator of childhood wellbeing [37].

**Figure 6:**
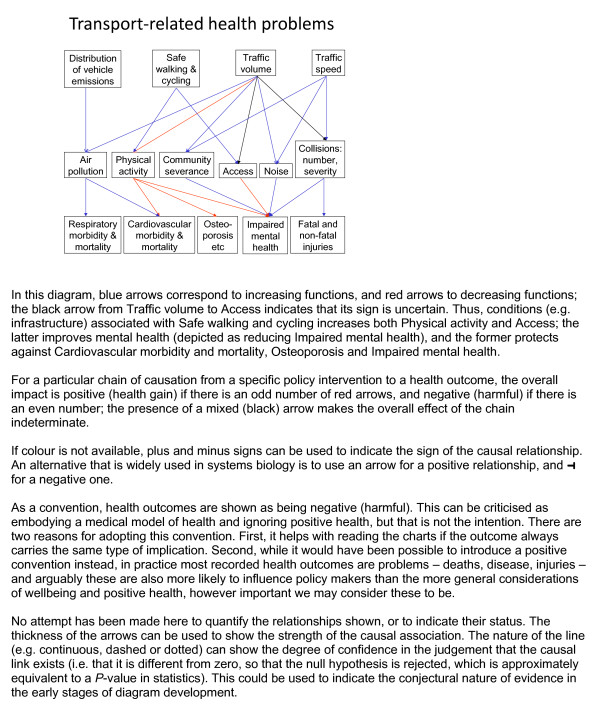
**An example of the web of causation**.

Moreover, a diagram with multiple and branching chains can readily be expanded to encompass a larger system, so enabling integrated analysis of the inter-related factors. In this case the upstream causes can include the wider determinants of ill-health as well as more concrete mediating factors - the "web of causation" for a particular health issue, a concept that has a long history [38,39] (see Figure 6 for an example).

By making the pathways explicit in a web of causation, a diagram deepens understanding and provides a framework for statistical analysis. In addition, it serves as a valuable practical guide: it not only provides multiple entry points for intervention, but also has the capacity to demonstrate and quantify the inter-relationship of different factors - including unpredicted and possibly undesirable side-effects. Strangely, although influence diagrams have been used informally to clarify hypotheses on the particular pathways that may be operating, it is rare to find causal diagrams being used as the basis for the statistical analysis of a system [40], as has been proposed in the context of setting out the evidence base for Health Impact Assessment [40] or Strategic Health Assessment [41,42].

**Figure 7:**
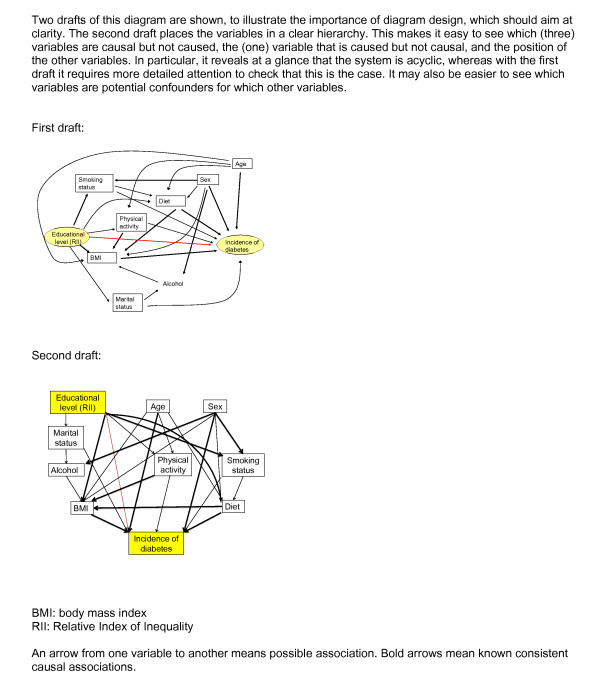
**A causal diagram used as the basis for statistical analysis**.

However, work along these lines is beginning to appear. Sacerdote and colleagues have used a causal diagram to organise the multitude of factors that are thought to influence the incidence of type II diabetes (Figure 7) [43]. And Rehfuess and colleagues have taken a similar approach to tease out the relative contributions of environmental and social factors that influence childhood death from acute lower respiratory infections in sub-Saharan Africa [44].

Modelling multiple and branching chains is more complicated than in the example of a whole-chain approach to exposure assessment as in Figure 5, because it involves the assumption that the chains are independent; in addition, intervention may involve multiple actions affecting more than one pathway, e.g. combining the use of "carrot" and "stick". Such diagrams are best organised by economic or policy sector; but the criterion for including variables and pathways in the diagram is that they are relevant to health - the content of the diagram is "driven by the bottom line" [40]. An additional layer can also be included below that for health outcomes, if so desired, on the economic costs of each of the adverse health outcomes. The analysis of a diagram of this type, and indeed confirmation of its structure, requires bringing together information from a number of different sources; and some aspects (such as "community severance" in Figure 6) may not be readily quantifiable. Multi-disciplinary research projects to integrate the relevant areas are currently underway [45].

## Properties and functions of causal diagrams

Causal diagrams are distinct from "mental maps", because they set out to describe relationships in the real world. The appropriate structure for a particular application is always driven by the content, so that the diagram is constructed by knowledge of the actual and possible pathways. For most people this is an intuitive and rather simple process, and informal diagrams have been used in non-academic situations, for example in stakeholder consultation in the context of Health Impact Assessment. In fact their flexibility and ease of use could lead to misuse, and one purpose of this paper is to make the case for the explicit further development of *rigorous *diagrammatic methods and associated statistical analysis.

A diagram can be used as the basis for a single study using a single dataset, but is not limited to this. As it conceptually maps out the research topic, it can have the function of synthesising the evidence from several distinct studies, including integration of multiple datasets that cover different parts of the causal web, and representation of qualitative as well as quantitative links. Thus, the diagram can be updated with new evidence as it accumulates.

A corollary is that a diagram can even be constructed when the evidence for some of the links is only tentative. The most important part is the structure, which is derived from substantive knowledge of a subject, as this is more difficult to modify later than the existence and strengths of individual component links. It may happen that more than one structure is possible, if different investigators have different conceptions of a system's causal relationships. This of course happens whether or not a diagram is used, and the advantage of using one is that it makes the different options explicit. They can then be discussed, and if appropriate, rival conceptions can be tested against the data. It is important that such a diagram is clearly indicated as being only conjectural; as evidence accumulates, the diagram can then evolve from having conjectural to well-supported status. Even at the conjectural stage, a diagram can have several important functions:

• to make assumptions and hypotheses explicit for discussion;

• to place hypotheses in the public domain prior to testing - a conjecture that is open to refutation;

• to plan data collection;

• to structure the statistical analysis of the hypothesised pathways;

• to identify evidence gaps and thereby to generate a research agenda.

Publishing the hypothesis of each study in advance of carrying out the research would remove the temptation for epidemiologists to adjust it once they have seen the data, which is an inevitable hazard of the rich datasets that are now available, and threatens to erode the distinction between hypothesis-testing and hypothesis-generating studies. This could conveniently be done in the form of a causal diagram, or more than one if disagreement is present between the researchers.

Depending on the degree of stability across different contexts, the application of a given model to different populations may require its modification. For instance, if the causal parameter for each component link varies between populations, and if its variation is systematic, the source of such variations can be included in the causal diagram, yielding a "hierarchical" structure.

**Figure 8:**
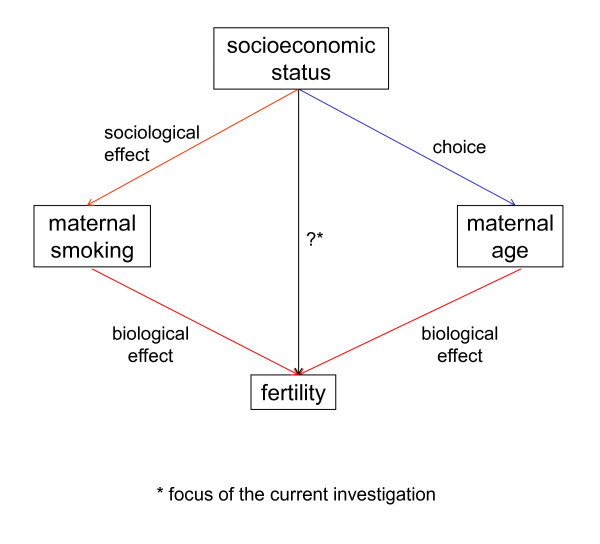
**Socioeconomic status and biological fertility**.

It may be thought that biological relationships are more stable than social ones, but this is not necessarily true: for example in the system depicted as Figure 8, the relationships of socioeconomic status with the distribution of age at the time of reproduction and with maternal smoking have been found to be highly stable, at least within western Europe in recent decades, at least as much as the biological pathways shown [46].

## Empirical aspects

Once a structure (or, rival structures) has/have been constructed, it/they can be used as the framework for statistical analysis of the component links. If in doubt, a postulated link should be included, as it can always be deleted in the light of evidence suggesting its magnitude is zero, whereas discovery of a link that was omitted in error is more difficult - although this can be achieved by algorithms incorporated in software e.g. in the context of DAGs used in genetics. The same applies to variables: they should be included, with all the pathways thought to be possibly relevant, unless and until analysis shows them to be unimportant. (This corresponds to how candidates for confounding variables are conventionally handled.) Thus the most conservative diagram contains all possible variables and pathways. The statistical analysis then results in deletion of some links, and quantification of those that remain.

In the deletion of links, it is clearly inappropriate to use a simple criterion such as striking out those that do not reach statistical significance. This is because a relationship could fail to reach significance merely due to small sample size. A better method is to use model comparison/selection methods such as those based on likelihood ratio (e.g. Akaike Information Criterion (AIC), or its Bayesian alternative the Deviance Information Criterion (DIC)). However, this process is fallible, especially in the presence of measurement error. An alternative is the use of structural equation modelling, in which latent variables can be introduced to deal with measurement error. The addition of a hierarchical layer modelling the relationship between observations and "true" values of a parameter could be considered, thus defining a hidden Markov Model [47].

**Figure 9:**
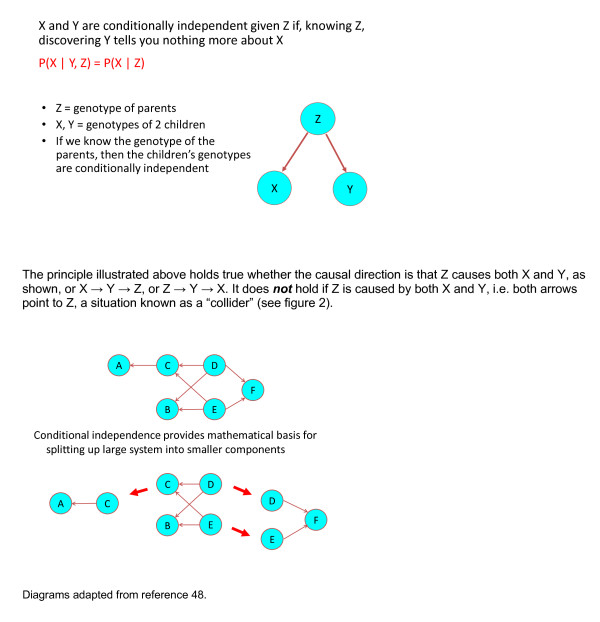
**Conditional independence**.

In some situations, a causal diagram can become large and complicated, and the quantification of its constituent links may rely on more than one dataset. The diagram then needs to be broken up into smaller components, with the risk of potentially introducing confounding or other distortions. However, this can be overcome if it is possible to use the conditional independence properties specified by the structure of the diagram: if two variables are connected to each other only via a third variable in one of the three ways depicted in Figure 2(a) - (c), then the first two are conditionally independent given the third one (see Figure 9)[48]. In statistical analysis they will be associated, unless the analysis adjusts for this third variable. These properties have been well understood within the graphical models literature for some time, and it is surprising that they have not already been widely exploited.

One of the distinctive features of a diagrammatic approach is that a causal pathway can be modelled using any parametric form, therefore separating the two key questions "does a link exist?", and "if so, what is its functional form?". This has an advantage over the specification of the system in terms of equations, where the elision of these two questions may be harder to avoid. For example, it is rather straightforward to draw a diagram such as that shown in Figures 6 or 7 from existing knowledge, but many of the causal relationships may be difficult to specify with any confidence. Another implication is that the use of causal diagramming clarifies the distinction between effect modification and statistical interaction; the latter may arise merely because e.g. linearity has been assumed in a situation where it does not correspond to the real functional form. Effect modification, on the other hand, corresponds to the situation where the relationship between two variables is altered by a third variable [34-36].

On the other hand, it is necessary to be cautious - diagrams may make the situation look simpler than it really is. An example of this is transmissibility: it may appear that if X → Y and Y → Z, then it is necessarily true that X → Y → Z. Logically it seems undeniable, but in real life this is not always the case. For example, in toxicology it is typically found that the dose-response relationship has a threshold: below a certain dose of the substance it has no impact on the organism. If this is represented by Y → Z, and the pathway X → Y does not result in the accumulation of Y to the threshold level, then X → Y → Z will not be true. This has fundamental implications even for basic data handling. For example, in studying the possible effects of disinfection by-products on the outcome of pregnancy, it was found that swimming led to infrequent but very high exposure levels [49]. If the exposure was coded as e.g. a weekly average, this was implicitly assuming that the actual exposure-response relationship is linear, which is not necessarily the case. The implication is that the assessment of one link cannot legitimately be considered separately from the characteristics of the neighbouring ones. This is easily missed if the inter-connections between links are not given their due weight.

## Extensions of the method

### Instrumental variables

One of the most intractable problems with epidemiology, other than in the (rather rare and special) situations where randomisation can be used, is that it is difficult to reliably infer causation from observational studies, because the upstream causal pathways are complicated and may introduce confounding or selection. One approach is to try and map out these pathways and analyse them in their own right. An alternative, which also involves analysing a system that is conveniently portrayed by means of a diagram, is to use the instrumental variable approach [50]. The basic idea is to find something that is outside the system being studied, and that influences the putative causal variable (actually "influences" here is misleading - the relationship does not have to be causal, only associational).

The principle is that one or more additional variables - "instruments"- are introduced, associated with the putative causal variables, but not directly with the outcome variable or any potential confounders. Further assumptions are that effect modification and alternative pathways are absent. All these assumptions need to be checked, and a convincing case made that they are satisfied; it is impossible for this to be conclusively established, a similar situation to the familiar case of unmeasured confounding. This approach is the equivalent in observational studies of analysis by intention to treat in randomised controlled trials.

A frequently-used method of statistical analysis has two stages: first, the instrument is used in a regression to obtain an estimated value of the putative causal variable, and then this estimated value is plugged into a second regression equation that contains the variables of substantive interest. The estimated value is an unconfounded measure if the above assumptions are met.

**Figure 10:**
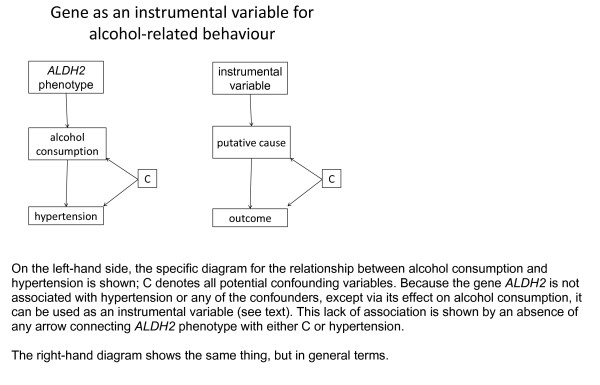
**Instrumental variables**.

In epidemiology, the main way that this has been introduced is mendelian "randomisation" (Figure 10)[51]. The idea derives from the fact that at meiosis (the cell division that produces eggs and sperm) there is a 50% chance which version of each gene gets through to the next generation. Whilst this is not strictly a form of randomisation, it is highly plausible that the gene is not directly associated with all the variables, e.g. social and behavioural factors, that are inter-related with the putative causal variable in a complicated way that is difficult to disentangle. Thus, the type of *ALDH2 *that a person inherits is strongly associated (causally) with the extent to which they enjoy heavy alcohol consumption, as already stated above, but is unlikely to be associated with the psychosocial factors that may be causes or effects (or both) of the level of alcohol consumption. The polymorphism for this gene can therefore be used as an instrument: it is a cause of the level of drinking that can be assumed to be independent of the various psychosocial and economic factors that would likely introduce confounding [52].

**Figure 11:**
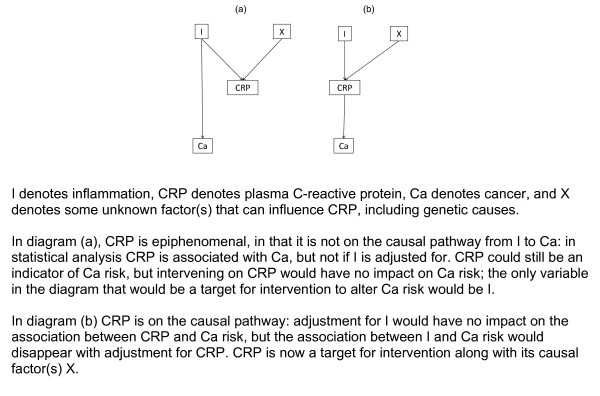
**C-reactive protein as a biomarker**.

This approach has been applied to the use of biomarkers: a biochemical measurement such as plasma C-reactive protein (CRP) can be a useful *predictor *of disease even when it is not on the causal pathway. But for *intervention *it is essential to know whether or not it is on the causal pathway; if it is merely epiphenomenal, then intervening to reduce it will have no effect. Figure 11 shows two possible scenarios, one in which CRP is epiphenomenal and one where it is on the causal pathway - a "mediator". Using a mendelian randomisation approach focusing on a combination of genetic variants that influence the level of CRP (as examples of X), statistical analysis showed associations of CRP with cancer (Ca) as well as with X. This is compatible with either diagram. However, no association was apparent between the combination of genetic variants and cancer, which strongly suggests (a) as the correct diagram - the convergence of two arrow-heads at CRP "screens off" such an association, whereas in (b) this association would be expected (unless CRP is conditioned upon). The conclusion is that CRP is merely an epiphenomenon [53]. A similar situation applies in the case of atrial fibrillation [54].

Such approaches can also be used in observational studies that do not involve genetics, as has long been routine in econometrics. A nice example is a study of the effect of family size on the mother's work status: to distinguish a direct causal effect from confounding (e.g. her preference for career as against childbearing) and from reverse causation (e.g. promotion leading to a decision not to have a further child, or not yet), the authors used the sex of the first two children as a natural experiment [55]. If they were of the same sex, the parents are more likely to want another child, for reasons unconnected with the labour market, so this plays the same role as deliberate assignment would if it were possible. Using this type of analysis in the context of a natural experiment could produce valuable evidence with a better grasp on the issue of causality than is often the case in observational epidemiology, but as far as we are aware this has not yet been attempted. An example might be the introduction of an alcohol tax that influenced consumption in an analogous way to the *ALDH2 *polymorphism - if the assumption is sustainable that there is no effect modification with other variables in the system.

### Change models

It is usual to construct diagrams in terms of the levels of the relevant variables, but an alternative is to instead use their changes. The mathematics of a change (or "first difference") model are different from one in terms of levels, a distinction that is very familiar in econometrics. One advantage is, any elements that remain invariant do not feature in a change model, so it can be a great deal simpler and thus more tractable. This invariance condition can be violated, for example in the presence of effect modification, or when the variable itself has a time-varying effect, such as the differing effect of maternal education on a child's IQ at different ages.

A second benefit is that interpretation is clearer: for example, it is relatively straightforward to think about the health impacts of a factory closure, whereas a discussion of the effects of (un)employment on health is more complicated, e.g. due to (self-)selection effects. Evidence derived from a change perspective may also carry more weight causally: for example, a controlled before-after study of a coal ban in Dublin showed the change in pollutant levels and in subsequent mortality there, but not in the rest of Ireland that was unaffected by the ban [56]. This is more convincing than when causation is inferred from cross-sectional studies [57]. In Bradford Hill's classic paper on inferring causation, he considered "experiment" - whether when "some preventive action is taken does it in fact prevent [the disease]?" - as "the strongest support for the causation hypothesis" [58]. However, caution is required: for example in the factory closure example, the health deficit that results is not necessarily the same as the health benefit that would occur in the reverse situation, i.e. if the same number of jobs were created (a possibility that is frequently put forward by proponents of capital projects, and which therefore is a recurring issue in Health Impact Assessment).

**Figure 12:**
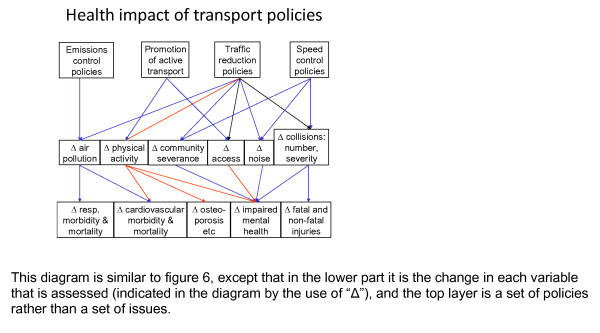
**A change model of the web of causation**.

An additional benefit of using a change model is that it fits naturally with a focus on intervention (Figure 12). Here the change in the upstream variables relates directly to a policy action. This issue is discussed more fully elsewhere [41,59]. It also relates to the previously-mentioned recommendation that natural experiments should be exploited more systematically, because such opportunities typically arise from policy interventions or other such changes.

### Feedback and cyclical models

**Figure 13:**
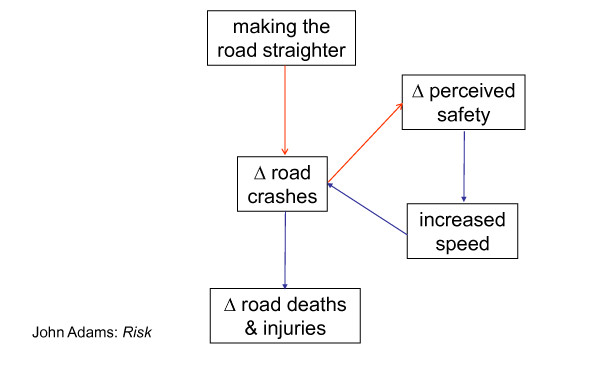
**A dangerous bend: risk compensation**.

Feedback may sometimes be important. A simple example is in the case of an "accident black spot": if the road design is improved so as to reduce the risk, drivers may respond by increasing their speed, thereby undoing some of the benefit - "risk compensation" [60] - an example of compensating (negative) feedback (Figure 13).

Reinforcing (positive) feedback may also occur, for example, people who are physically inactive may tend to become obese and have other physical changes that further discourage them from exercising, and conversely, more active people have physiological changes that encourage them to take more exercise (this feedback mechanism seems plausible although there is no clear evidence for it). Reinforcing feedback also frequently occurs in models of population growth, and therefore in infectious disease modelling which is derived from demography and ecology. For example, this occurs when parasites sexually reproduce (such as worms that cause chronic tropical diseases): as the parasite population size increases, assuming that the sex ratio remains fixed, so does the likelihood that males and females will discover and mate with one another and therefore population growth occurs faster [21].

More generally, as humans tend to respond systematically to their situation, if the response (e.g. policy or other intervention) is included in the model, then feedback may have to be taken into account. This is true also of conditions (or social issues with health consequences) that involve a large behavioural element, such as obesity, mental health and homicide [61].

In general, analysing systems with feedback requires a different approach, with diagrams that contain cycles. In infectious disease epidemiology, this is explicit because feedback loops are the rule and acyclic diagrams the exception. Infectious disease epidemiology models are, in general, system dynamical in this sense, and off-the-shelf software such as *Vensim *[62] is often used to construct models. An excellent account of the issues and methods, in the context of business studies, can be found in Sterman [22]. An important feature of systems containing feedback is that they tend to have the property of generating their own endogenous causation processes [63,64], a simple biological example being homeostasis, a compensating feedback system that keeps a variable such as potassium or cortisol concentration, or temperature, at an appropriate level.

**Figure 14:**
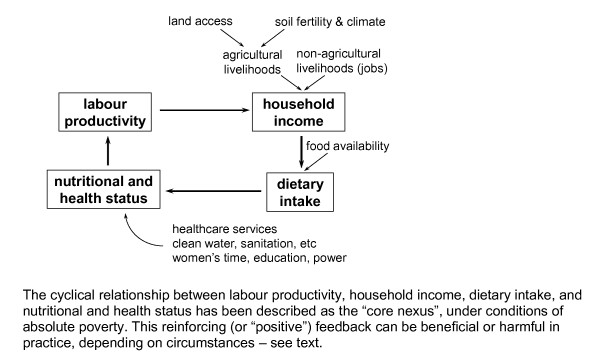
**The situation of a household under conditions of absolute poverty**.

From the viewpoint of intervention, it is important to recognise when feedback is occurring, particularly because the usual methods of thinking, epidemiological and other, tend not to see it. Thus, compensating feedback frequently occurs, like the risk compensation example, and frustrates policy interventions. And reinforcing feedback - often dismissed pessimistically as a "vicious circle" - can be an opportunity: in the context of absolute poverty, health influences the economic wellbeing of a household, and the latter in turn is a "fundamental determinant" of health [65]. The implication is that an intervention in one area, e.g. that prevents malaria, or that improves agricultural productivity, can have impacts beyond the immediate target, that are transmitted around the cyclical diagram (Figure 14)[65].

## Conclusion

Explicitly causal methods of diagramming and modelling have been greatly developed in the past two decades. However, use of such methods in epidemiology has been mainly confined to the analysis of a single link: that between a disease outcome and its proximal determinant(s). Apart from in the context of infectious diseases, they have been under-exploited in their potential to model the larger system in which health is generated or undermined.

This approach would accord with wider developments in biology. The Human Genome Project has revealed that the number of protein-coding genes is far fewer than was previously thought, and that they are influenced by upstream genes in that large proportion of DNA that was previously referred to as "junk" [66]. "Causes of causes" are therefore relevant outside the realm of epidemiology. In addition, more complicated networks, consisting of multiple and interacting causal chains, sometimes with regulatory feedback, are the focus of the increasingly important interdisciplinary field of systems biology [67].

Diagrams and models are constructed to fit each situation, from a combination of substantive knowledge and statistical evidence - but can then take on properties that result from their abstract structures. By constructing diagrams of a larger system, inter-relationships of different factors can readily be visualised, and then analysed statistically. As well as its scientific function, this has practical advantages in terms of designing interventions.

Such methods are applicable to all branches of epidemiology, including infectious diseases epidemiology, chronic disease epidemiology, environmental and occupational epidemiology, and social epidemiology - and especially to their inter-relationship, e.g. simultaneous consideration of social and environmental influences.

## Competing interests

The authors declare that they have no competing interests.

## Authors' contributions

All the authors contributed substantially to the writing of the paper, the drafting and the process of critical revision. The main drafting was carried out by MJ, with important contributions by MG, MC-H and PV according to their particular range of expertise. All authors have read and approved the final manuscript.
